# New Appliances and Things Medical

**Published:** 1910-01-08

**Authors:** 


### NEW APPLIANCES AND THINGS MEDICAL.

[We shall be glad to receive at our Office, 28 & 29 Southampton Street, Strand, London, W.C., from the manufacturers, specimens of all new preparations and appliances.]

### GLOBE METAL POLISH.

We have received from Messrs. Raimes and Co., Ltd., Bow, London, E., who are the sole agents, samples of the above preparation. The Globe Liquid Metal Polish, supplied in convenient sized tins, is one of the best of its kind we have seen. It gives a brilliant, clean, sparkling polish, is non-corroding, and free from any unpleasant smell. Equally excellent is the Globe Extract Metal Paste, which is perhaps more easy to apply successfully. We have used both varieties for cleaning surgical instruments, and metal ware of various descriptions, and have found them decidedly useful and in many respects superior to most of the metal polishes on the market. The practitioner will find it worth while to order a tin of the polish paste and keep it handy in his surgery. The comparative cheapness of the polish is no index of its extreme usefulness and excellence.

